# Developing a Sense of Knowing and Acquiring the Skills to Manage Pain in Children with Profound Cognitive Impairments: Mothers' Perspectives

**DOI:** 10.1155/2017/2514920

**Published:** 2017-03-26

**Authors:** Bernie Carter, Janine Arnott, Joan Simons, Lucy Bray

**Affiliations:** ^1^Faculty of Health and Social Care, Edge Hill University, Ormskirk, UK; ^2^School of Health and Well-Being, University of Central Lancashire, Preston, UK; ^3^Faculty of Health and Social Care, The Open University, Milton Keynes, UK

## Abstract

Children with profound cognitive impairment (PCI) are a heterogenous group who often experience frequent and persistent pain. Those people closest to the child are key to assessing their pain. This mixed method study aimed to explore how parents acquire knowledge and skills in assessing and managing their child's pain. Eight mothers completed a weekly pain diary and were interviewed at weeks 1 and 8. Qualitative data were analysed using thematic analysis and the quantitative data using descriptive statistics. Mothers talked of learning through a system of trial and error (“learning to get on with it”); this was accomplished through “learning to know without a rule book or guide”; “learning to be a convincing advocate”; and “learning to endure and to get things right.” Experiential and reflective learning was evident in the way the mothers developed a “sense of knowing” their child's pain. They drew on embodied knowledge of how their child usually expressed and responded to pain to help make pain-related decisions. Health professionals need to support mothers/parents to develop their knowledge and skills and to gain confidence in pain assessment and they should recognise and act on the mothers' concerns.

## 1. Introduction

Children with profound cognitive impairment (PCI) can experience pain from a wide range of different sources. Some of these pains are the commonplace pains of childhood (e.g., toothache) but some are associated with their underlying disorder (e.g., muscle spasms, gastrooesophageal reflux) or with the child's impairments and the prescribed treatments and interventions (e.g., venepuncture, pain related to use of splints) [1, 2]. Alongside this array of different types of pain, children have a wide range of responses. The heterogeneity of response to and expression of pain in this diverse group of children may be related to the child's comorbidities and motor development disabilities [3] and the effect these may have on their physiological and behavioural responses (Breau et al., 2001). This in turn can result in ambiguity and misinterpretation of pain-related behaviours [4]. The combination of these factors can result in a “perfect storm” whereby children with PCI experience frequent, persistent, significant, and sometimes daily pain [5–8] and are at high risk of their pain being underassessed and undertreated [4, 9, 10].

Specific pain assessment tools are available for use with children with PCI but there is limited evidence underpinning these tools' reliability, validity, and clinical utility [11]. However, there is evidence to show that using a tool specifically designed for this group of children results in more precise assessment of their pain than using a generic pain assessment tool [12]. Appropriate tools, recommended for use within this population in the UK by the Association of Paediatric Anaesthetists [11] include the Paediatric Pain Profile [13], the revised-Face, Legs, Activity, Cry, Consolability (r-FLACC) tool [14], the Noncommunicating Children's Pain Checklist-Revised [15]. The r-FLACC has been shown to have the most clinical utility for health professionals [16], not least because it is “quick to complete.” There is robust evidence that pain tools are embedded inconsistently within practice settings [17] and other literature reveals that health professionals perceive the specialist assessment tools to be more complicated than the more commonplace ones used for acute pain assessment in nonimpaired children [2, 18]. Consequently, health professionals have reported uncertainty and a lack of confidence in assessing pain in children with PCI [19, 20]. In the face of uncertainty about pain assessment in children with cognitive impairment, health professionals often turn to parents of children with PCI for guidance, who, regardless of their sensitivity to their child's pain [21], underestimate it as well [22]. As a result, pain assessment in this heterogenous group of children poses challenges for parents and health professionals [1, 23]. Children with PCI share the characteristic of being unable to self-report their pain; this shifts assessment from the “gold standard” of self-report to reliance on proxy identification of pain, usually by the child's parents [24] who rely on behavioural indicators of pain [11, 25]. Parents need time to develop knowledge, skill, and judgement in knowing whether their child is in pain [2]; they also require access to information to promote their confidence, accuracy, and advocacy skills [26, 27]. Most studies on pain assessment in children with PCI focus on the development and validation of pain assessment tools [13–15] or implementation of these tools into practice settings [26] but do not examine how parents and health professionals develop and acquire knowledge and skills in assessing and managing pain in children with complex needs. This is a significant omission in the literature as it means that health professionals have little understanding of the approach, timing, and pace required to support parents of children with PCI to acquire the requisite skills and knowledge. The current situation means that children with PCI are likely to experience suboptimal pain assessment and pain management resulting in children experiencing the physical and emotional consequences of poorly managed pain.

This paper reports on parent-generated data from a study that also addressed health professionals' experiences and perceptions of assessing and managing the pain of children with PCI.

## 2. Methods

We aimed to explore the frequency, regularity, and intensity of parent-reported pain episodes experienced by children with PCI and their parents' knowledge and skills in assessing their child's pain.

### 2.1. Design

We adopted a convergent parallel mixed method design [28], using quantitative (survey) and qualitative (interview) data collection methods over an eight-week period. A mother and father each with a child with PCI and regular episodes of pain provided invaluable advice and input at the design and early stages of the study.

### 2.2. Sampling, Inclusion, and Exclusion Criteria

We used purposive sampling with the aim of recruiting mothers and fathers providing daily care of children aged 2–16 years from tertiary children's hospital. The children were identified by clinicians as having PCI, were unable to self-report pain, and had experienced at least one episode of pain in the previous month. We devised a sampling matrix to facilitate recruitment of equal numbers of boys and girls within two age bands (2–8 years, 9–16 years). Our underlying assumption was that parents would continue to be challenged by and learn about their child's pain so the age range was selected to engage with parents at the early stages of developing and acquiring knowledge and skills as well as those consolidating their knowledge and skills in managing their child's pain. Each child's parents were eligible to participate. Children were not eligible if they had a primary diagnosis of an autistic spectrum disorder (ASD) (as their communication difficulties are of a different type to our proposed target group), where there were known safeguarding issues within the family, where the treating clinician deemed it inappropriate to approach the family and where the parent(s) level of English language would prevent participation in the study.

### 2.3. Ethics

Ethics approval was gained via the NHS Research Ethics Service (14/NW/0106) and through the tertiary children's hospital. Informed consent was gained from each participant and ongoing consent was checked verbally at each point of contact. A referral protocol was in place to deal with any issues that raised researcher concerns (e.g., safeguarding, uncontrollable pain). All relevant governance protocols relating to data management and pseudo-anonymisation were followed.

### 2.4. Data Collection

Surveys and interviews were the selected methods of data collection.

#### 2.4.1. Pain Survey

We used an established semistructured survey [5] consisting of a mix of open (*n* = 7) and closed (*n* = 8) questions to generate data on the number of reported pain episodes experienced by the child, the perceived cause, intensity, duration, and timing of pain episodes, and the participants' response to those episodes. The survey was administered weekly for eight weeks. Participants were given the option of the survey in weeks 1 and 8 being face-to-face or by telephone; weeks 2–7 were only undertaken by telephone. We planned to account for each child's circumstances and participants could pause, withdraw, or continue their engagement in the study at any time.

#### 2.4.2. Interviews

We used both unstructured [29] and semistructured audio-recorded face-to-face and telephone interviews [30] to elicit their subjective accounts of their experiences of and insights into their child's pain. The week 1 interview was unstructured with the aim of enabling participants to tell their own stories about their child's pain, including how they acquired their skills and knowledge. The week 8 interview was more focused giving participants the opportunity to reflect on whether their engagement in the study had any effect on their skills, knowledge, or approach to their child's pain or their engagement with health professionals. Face-to-face interviews took place in the child's home and at a mutually agreed time. The option of a telephone interview was available where this was either preferred or more convenient.

### 2.5. Data Analysis

Interview data and data from the open responses from the survey were analysed using thematic analysis [31]. Each participant's dataset was analysed individually before considering all the transcripts as a complete dataset. Each member of the research team undertook coding and memoing of selected interviews. We worked iteratively moving between transcripts and codes to identify emerging themes; the use of multiple coders added to our iterative and interpretive approach and helped to promote the quality and rigour of analysis [32]. Discussion between the researchers took place until a broad understanding and consensus about initial themes was achieved and we had attended to negative cases. Further coding and iteration resulted in the generation of a metatheme and three core themes. The closed questions from the survey were analysed using simple descriptive statistics and these data (e.g., frequency, regularity, and intensity are presented in Table 1).

#### 2.5.1. Findings

Eight mothers of children with PCI participated in the study. All of the children in the study lived at home with their family. No fathers chose to be interviewed although those who were in the home during an interview provided confirmation of or added to the mothers' description of the challenges they faced. As they had not given consent at the start of the interview and were not available by the end of the interview we did not attempt to collect retrospective consent and thus cannot directly report their contributions. However, these informal contributions by the fathers affirmed the mothers' responses and often added emphasis to what the mothers were reporting. The children of the participants were 7–16 years of age; four were boys and five were girls with a range of different diagnoses. Two children were reported as having one source of pain; four children had four sources, one child had five sources, and two had six sources of reported pain during the 8-week period. Most had a high pain burden with worst pain scores ranging from 4 to 10 (mean score of 8). All children we have data for experienced pain on a regular basis; six children experienced pain either daily/nightly or most days. Four children had pain related to spasms associated with feeding, bowels, and “gripe” [colic]. Some pains were troublesome and resistant to treatment, often requiring more than one medicine. The mothers used a range of nonpharmacological strategies to make their child as comfortable as possible. All of the mothers talked of being confident about aspects of assessing managing their child's pain but were also challenged by the complexity of the pain, the need to protect their child, and for one mother the fact that pain was now a* “bigger issue than all other symptoms”* (M2) (see Table 1).

### 2.6. Metatheme: Developing a Sense of Knowing

**Figure 1:**
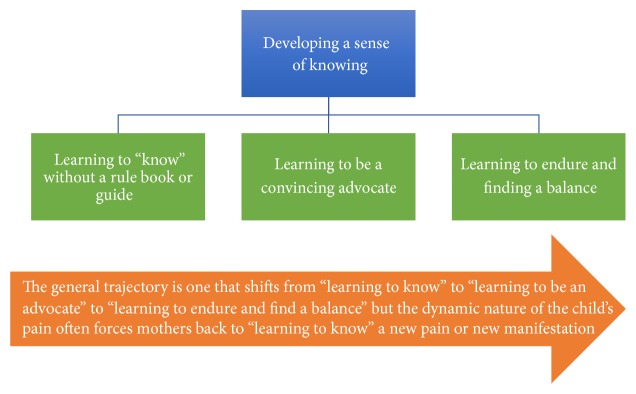
Metatheme and core themes.

“Developing a sense of knowing” describes the ways in which the mothers had to learn how to assess and manage their child's pain through a mix of trial and error resulting in an intuitive sense of knowing what was wrong with their child. This acquisition of skills often occurred in their own homes and with little formal support from health professionals. This metatheme is composed of three core themes “learning to know without a rule book or guide”; “learning to be a convincing advocate”; and “learning to endure and finding a balance” (see Figure 1).

#### 2.6.1. Learning to “Know” without a Rule Book or Guide

The mothers learned to “know” their child's pain, although not necessarily the cause(s), through their constant presence in their child's life. Their closeness to their child meant that they developed an awareness of the sometimes subtle and nuanced changes that occurred when their child was experiencing pain. Some mothers saw this as an extension to their parenting role talking of it as* “informed guesswork, but that's motherhood generally”* (M2). However, the level of skill that the mothers developed with their children with PCI went beyond what would be expected of “ordinary” parenting. Developing this* “sense of knowing”* took time to become embedded and the mothers did not take this knowledge, described as* “a sixth sense or a gut feeling”* (M2), for granted. Participants recalled the early days when they were overwhelmed as* “you just don't have a clue what to look for or what to do”* (M4).

“Knowing” was often based on* “just trying to work out where the pain is ‘cos he can't tell you'”* (M1) and required mothers to pick up and “read” the constellation of behavioural indicators and cues that provided them with the evidence that their child was in pain. These cues were wide ranging and included noises as follows:* “he makes that gurgling noise”* (M1),* “he goes ch-ch-ch-ch-ch”* (M4),* “he'll start screaming and crying”* (M3), or* “tone of his voice”* (M6). Other cues included facial expression* “her face is like a book”* (M2); mood changes* “she was just inconsolable”* (M5); and changes to posture and movement* “she'd hold herself quite stiff and rigid”* (M6),* “he draws his legs up”* (M4), or skin colour* “almighty red blotches”* (M6). The mothers were able to describe specific behavioural characteristics; the “look” on their child's face or the* “look in their eyes”* was often referred to; for example,

you can see by her face she's like… a bunny in the headlights… she has this terribly startled, shocked look. (M2)

The dynamic nature of their child's underlying condition, comorbidities, and/or changing interventions meant that their child's pain burden was also dynamic with new and sometimes unexpected causes of pain occurring. Confidence and certainty could be undermined when the child's pain changed. One mother explained:

…you never actually stop learning things from your child, you're constantly, adapting to the next situation, the next problem and interpreting it… acting on it. (M2)

The mothers wanted to learn and know more about what was causing their child's pain and what to do about it. However, they were aware that on occasions the health professionals were also unsure about the cause and most appropriate course of intervention or management. The mothers talked of having to learn to manage their child's pain without a* “rule book”* and some felt that they had to cope without anyone to act as a guide. Their perception of the situation was that they were often journeying* “on their own”* (M3), “*left to paddle your own canoe” *(M2), or* “taking each day as it comes” *(M7) as they tried to respond to their child's ongoing pain. They talked of health professionals being unable to provide guidance and support as their child was* “different to other children”* or as one mother explained* “she's a little work in progress” *(M6).

As they developed expertise and insight, they created their own rule book for their child and some mothers could accommodate their child's changing pattern of pain:

He's like a Rubik's cube because you're constantly adjusting things and adding things and taking things away to make sure he's alright and you do it all the time, twenty four hours a day… he is difficult! (M3)

However, some mothers became somewhat overwhelmed by the expertise they needed to develop to accommodate the increasing complexity of their child's condition, pain burden, and polypharmacy. One child had a pain plan indicating what medications could be given if breakthrough pain occurred or predictable pain situations occurred. This plan was in place to help ensure that the child's pain treatment was streamlined, proactive, and effective; this was highly valued by the child's mother.

#### 2.6.2. Learning to Be a Convincing Advocate

One of the frustrations that the mothers expressed was that whilst they knew their child, the health professionals tended to look through a more fragmented lens. They often described the health professionals as being less comfortable in* “intruding”* on other specialists' territory and* “not very good at joining the dots”*:

The specialists just look at the bit they're interested in; it's up to me to put it all together. The neuro are interested in epilepsy, and the gastro in his stomach but no-one is really responsible for tackling his pain. (M3)

This fragmented, systems-oriented approach inevitably meant that the child's pain sometimes slipped through the cracks between the specialisms; this was particularly frustrating for when neither the mothers nor the specialists could identify the source of the child's pain.

As mothers developed their own skills and learned to trust their own judgement about their child's pain expression and behaviour they sometimes also faced the additional challenge of convincing sceptical health professionals that their child was in pain. Mothers often found it difficult to deliver the level of “proof” they thought that health professionals wanted. They had to act as both interpreters and translators of their child's pain: interpreters in that they could interpret their child's pain cues, behaviours, and responses and translators as they were able to explain to other people that their child was in pain. The mothers valued health professionals who would listen to them and carefully consider the proxy evidence that they presented and who did not dismiss their concerns about their child's pain because they were* “just a parent” *(M4). One mother explained that* “I don't claim to be a doctor…. but I know my own baby” *(M5). Another mother explained that she was now very confident and able to “*tell them what's wrong with him” *and could guide consultations, commenting further that* “without sort of blowing my own trumpet – I'd say 80% of the time we're [mother and father] right” *(M4). The mothers talked about how self-belief helped them to act as an advocate for their child. Some mothers talked of how they had been taught to use the PPP and how this could provide health professionals with additional and more concrete evidence of a child's pain, especially when* “she scored really high” *(M6).

#### 2.6.3. Learning to Endure and Finding a Balance

Most of the children had frequent, ongoing episodes of persistent pain that were sometimes described as* “overwhelming”* (M2) and very distressing for mothers, although these episodes of pain were rarely witnessed by health professionals. This ongoing pain required both mothers and their children to endure as one mother explained:

you see them suffering horrendously…you know in agony, not sleeping, not eating, not drinking because they're in so much pain. (M1)

The persistence of pain and its resistance to medication meant that the mothers felt like they had to* “battle”* (M3) and* “do everything”* (M4) to get adequate analgesia to manage their child's pain even though “*it might not even be touching the tip of the iceberg of her pain” *(M2). One mother talked of her son* “fighting”* (M7) his pain. One mother stated when nothing manages her child's pain that she* “just ride[s] with it” *(M5); whilst this seem to be a passive response, there is a point at which “riding the pain” may be the only way to go.

Some mothers talked of the balancing act they performed between managing their child's pain and the adverse consequences of pain medications such as heavy sedation on their child's ability to engage in family life, explaining* “what life is that?” *(M7). Some talked of a reluctance to use* “strong”* medicines that inhibit breathing or have other adverse effects explaining that they would* “try and hold off” *(M7) these medicines or only use them* “as a last resort” *(M8).

Others talked of trading one thing against the other and sometimes rejecting medical advice. One mother explained that she wanted to continue to give her daughter regular paracetamol and ibuprofen as it meant she could bathe and dress her in the morning without causing her pain, despite* “the powers that be [doctors] saying she shouldn't need them now”*; she further explained:

She's in less pain when she's in bed but that's not a life, just being in bed. She needs to be part of the family, up in her chair and being with us. That's a trade off. (M2)

Another mother explained that rather than following the regime suggested by the doctors to give her son regular sedation (chloral hydrate) through the day to manage the* “wretching *[*sic*]*… arching… screaming”* pain associated with his tube feeds, they had chosen, despite massive disruption to her sleeping patterns, for him to have his feeds overnight* “so he can be part of the family during the day.”* The mothers were also aware of the consequences of their child taking medication and were insightful about the potential long-term effects of drugs. This was especially the case if the drugs were not optimally effective as one mother explained* “if we're just numbing it, then he can't be on it for life” *(M1).

## 3. Discussion

The children in this study had a high pain burden; they experienced frequent, intense pain from a variety of sources and the incidence is broadly like other studies of this population [5, 7, 8]. Parents were clearly challenged by their children's pain, regardless of the source or type of pain, and many of them talked in terms of pain management being a “struggle” although like parents in other studies they were able to articulate their child's pain responses [9]. Despite this most of them reported feeling confident in knowing whether their child was in pain.

A strong thread of experiential and reflective learning was evident in the way the participants talked about how they had developed a “sense of knowing” about their child's pain. Arguably, this is how all parents learn their parenting skills. However, for the parents of children with PCI, there are no reference points from which to start to piece things together. There are many different perspectives on and theories of experiential learning [33–35]. At its most simple meaning, experiential learning is about “constructing knowledge and meaning from real life experience” (p161) [36]. Fundamental to experiential learning are three explicit assumptions: “learning is ‘situated'; it can be viewed either as an individual or collective process; and it is triggered by authentic practice based experiences” (pe102) [37]. Experiential learning is also inherently participatory [36].

Evidence of the success of this experiential learning was the deep and intrinsic knowledge the mothers had of how their child usually expressed and responded to pain. The range and extent of pain the children experienced is in line with findings from other studies [5, 6]. The mothers were exposed to ongoing and deeply authentic learning experiences; confronted by their child's pain, they had little choice but to engage and learn. All the participants' learning had been situated within authentic real-life learning environments [38]; none of the learning was simulated. They were learning by making real judgements in the home setting about whether the child was in pain. The mothers had no choice but to engage, to learn, and* “to learn quickly”*; their child depended on them to* “get things right.”* They had to learn the assessment skills they needed, develop the judgement required, and reflect on past experiences to make meaning out of their child's pain behaviours and ways of expressing pain. This learning was ongoing and contextual and for the most part it occurred without any form of facilitation from an expert. Considering how little support the mothers had, especially compared to the supportive curricula that surround most health professionals' learning, it is impressive how knowledgeable and skilled they became in many of the pain situations they faced.

The mothers demonstrated their “innate capacity to grow and learn” [39] and on many occasions within the interviews they surprised themselves by reflecting on how far they had come from the early days where they had been overwhelmed. They learned advocacy skills to present concerns about their child's pain to health professionals. As time went on, the mothers could act more effectively as an advocate for their child and they enacted their advocacy role by being prepared to take on the perceived power of health professionals by becoming more fully involved in decision making. For some parents their advocacy was enhanced, as seen in other studies, through the security of a knowledge base [26] supported through using a validated pain assessment tool: the PPP [13]. As their confidence grew the asymmetries in traditional authority and power [40] became less of a concern and they felt more sure about making decisions, for example, about trading the benefits and adverse effects of pain medicines. Their learning had been individually transformative in terms of them feeling more confident in their knowledge with a grounded “gut feeling” about their child's pain. Their learning occurred partly because they were attuned to their child through being a constant presence [2, 20] and although they did not use the word “reflection,” it was clear that they attended to their own feelings about what they thought was happening with their child. Attending to feelings is an aspect of embodied knowledge and whilst the mothers talked of gut feelings, this appears to be them “trust[ing] their bodies as a site of knowing” [41]. Taking this concept of embodied knowing further it is interesting to speculate that this was perhaps dualistic, not just trusting their own embodied knowledge but also being so close to their child that they could trust their child's body (through touch, sight, and insight) to complement their own embodied knowing of their child's pain. They learned to recognise and interpret the subtle changes in their child's behaviour and modes of expression becoming attuned to their child's idiosyncratic pain cues. Their knowledge of their child's specific pain cues resonated with the evidence base for the diversity of pain cues exhibited by children with PCI [2, 42] and with those cues used various pain assessment tools such as r-FLACC [14].

However, there was still a sense of* “trial and error”* and* “experimentation,”* especially when a new pain occurred or when an existing pain became resistant to intervention. As seen with other parents, the mothers in this study needed time to acquire this knowledge [20]. Whereas ideal experiential learning occurs in a supportive environment facilitated by experts to help the learner make sense of their experiences, the mothers' learning was most often solitary without the facilitative dialogue of experts [43] that has been shown to promote meaning-making. In situations of uncertainty, participants sought help from health professionals and, at these times, they wanted the health professionals to listen to and “believe” them. This did not always happen. So rather than a constructive dialogue [37] that could have engendered learning for both the health professional and the mother, the outcome was sometimes a stilted, diminishing, and frustrating experience. Where mothers perceived that health professionals recognised their knowledge, even when this knowledge was embodied and difficult to translate into clear “clinical talk,” they felt rewarded, respected, and more confident.

### 3.1. Limitations

We acknowledge that the inclusion criteria were broad and this can be viewed as a limitation as we recruited a heterogeneous sample of children in terms of age, diagnosis, source of pain, and other factors. Our sample size is small. We also have missing survey data for two children so our data set is incomplete.

### 3.2. Practice Implications

Most mothers learn about and make meaning of their child's pain cues without the facilitation of experts. Mothers have to manage their child's high pain burden, often on a daily basis. Much of mothers' knowledge is embodied and hard to articulate and predicated on a sense of “knowing.” An appreciation and awareness of the subtle, individually responsive, reflective, and progressive way in which mothers learn how to assess and respond to their child's pain can help health professionals to provide them with better support. Mothers become experts in their child's pain, but this is an expertise they did not choose; it is a journey of discovery. Health professionals can help support parents by recognising and acknowledging this expertise whilst continuing to actively support parents along that journey of discovery. Exploring what individual mothers perceive to be “effective pain management” and a “good family life” can help establish a common ground between parents and health professionals about expectations and reduce feelings of isolation. Responding to mothers' genuine concerns about their child's pain, medication, and the adverse effects of medicine is important in enhancing the quality of clinical care provided to children with PCI. Mothers can positively contribute to that care but may need support and space to do so.

## 4. Conclusion

Experiential learning is fundamental to the ways in which the mothers learned to assess and manage the pain of children with PCI. Mothers draw on embodied knowledge to help make decisions and they are insightful about the fact that as their child's pain changes they will continue to be challenged, need to adapt, and learn more. Their in-depth knowledge gives them confidence to act as an advocate for their child. They demonstrated sophistication in the way they trade pain management and the adverse effects of the drugs with the importance of their child having as good a life as possible and being part of the family.

## Figures and Tables

**Table 1:** Overview of children, pain experienced, medication, and actions taken during the period of data collection.

Mother	Child	Sources of pain (using participant descriptors)	Frequency and regularity of child's pain	Intensity of worst pain (0–10 scale) & nature of worst pains (as scored by participant)	Pain plan	Medication given by participants to manage pain	Other (nonpharmacological) actions taken	Level of challenge, confidence, expectation in relation to managing pain
M1	Boy, 11 yrs, birth asphyxia, severe epilepsy, stomach ulcers	(i) Stomach migraine (ii) Headache (iii) Abdominal pain (iv) Hip pain (v) Pain of unknown origin	(i) 8 out of 8 weeks (ii) Daily	(i) Intensity: 8 (ii) Nature: pain causing drawing up of knees	No	Acetaminophen	(i) Rubbing back and legs (ii) Positioning (iii) New bed and wheelchair	(i) *Challenge:* struggling with ongoing pain (ii) *Confidence:*confidence is low with unknown cause of pain and uncertain if actions are working(iii) *Expectation:* not stated

M2	Girl, 11 yrs, severe neurological impairment, epilepsy, postscoliosis surgery	Nerve pain following scoliosis surgery	(i) 8 out of 8 weeks (ii) Daily	(i) Intensity: 9 (ii) Nature: nerve pain and spasms in back, left leg, and arm	No	(i) Acetaminophen (ii) Ibuprofen (iii) Baclofen (iv) Gabapentin (v) Midazolam (rescue medicine)	(i) Positioning (ii) Bath (iii) Massage	(i)* Challenge: *an ongoing struggle; pain is now a bigger issue than all other symptoms (ii) *Confidence: *informed and confident she will do her best but lacking confidence in professionals accepting responsibility for pain management (iii) *Expectation: *it is up to her to fight for better pain management; hope that current cycle of pain will be resolved

M3	Boy, 7 yrs severe neurological impairment, feeding difficulty, epilepsy, cardiac problems	(i) Stomach spasms (with night feeds) (ii) Hip pain (dislocated) (iii) Dystonia (iv) Muscle pain (from foot splints)	(i) 8 out of 8 weeks (ii) Every night	(i) Intensity: 10–12 (parent knowingly stated that pain could be 12 out of 10, as it “went off the scale”) (ii) Nature: “unrelenting” pain (tube feeding); acute pain on movement (hips)	No	(i) Chloral hydrate (ii) Fentanyl (patches)	(i) Settling him to bed (from chair) (ii) Winding [burping] him before bed and settling	(i) *Challenge:* struggling with ongoing pain and the impact this has on her and her family (ii) *Confidence:* confident in herself and her ability to assess her son and manage him to the best of her ability (iii) *Expectation:* he has always had pain and he will always be in pain

M4	Boy, 10 yrs, severe brain damage, quadriplegia, asthma, sleep apnoea, double hip reconstruction	(i) Stomach cramps/indigestion (ii) Gastritis (iii) Constipation (iv) Hip pain (v) Toothache (vi) Helicobacter (stomach pains)	(i) 8 out of 8 weeks (ii) Most days	(i) Intensity: 6 (ii) Nature: spasm type pains associated with stomach and bowels, nagging pain from hips	No	(i) Acetaminophen (ii) Botulinum toxin Type A (iii) Gabapentin (iv) Enemas (v) Probiotics	(i) Diet (lactose-free) (ii) Positioning (iii) Startling him to “break cycle of pain”	(i) *Challenge:* managing reasonably well although pain is ongoing (ii) *Confidence:* confident, well organised, and supported by carers; able to stand ground with professionals and make things happen (iii) *Expectation:* he will always have some pain but that it will be able to be managed reasonably well

M5	Girl, 9 yrs, severe brain damage, epilepsy, cerebral palsy, scoliosis, sleep apnoea, tracheostomy	(i) Muscle spasms (ii) Stomach cramps (iii) Constipation (iv) Coughing	(i) Not available (ii) Regularity (not available)	(i) Intensity: not available (ii) Nature: spasms (muscles) and gripe	No	(i) Acetaminophen (ii) Baclofen	(i) Positioning (ii) Distraction (talking, stories) (iii) Bath	(i) *Challenge:* managing medications to ensure child has “good nights” (ii) *Confidence:* confident that she can work out what is causing child's pain (iii) *Expectation:* not specifically stated

M6	Girl, 9 yrs, cerebral palsy, scoliosis, dislocated hip	(i) Hip (ii) Spine (iii) Reflux (iv) Fractures	(i) 6 out of 8 weeks (ii) At least once a day; often continuous	(i) Intensity: 4–6 (ii) Nature: positional, broken leg, breathing, and chest	Yes	(i) Acetaminophen (ii) Morphine	Being in comfortable bed	(i) *Challenge:* child “keeps fracturing,” ongoing challenge (ii) *Confidence:* able to juggle medicine up and down as needed due to experience (iii) *Expectation:* need to be constantly alert to likelihood of child being in pain

M7	Boy, 7 yrs, meningitis, hydrocephalus, cerebral palsy, epilepsy, Crohn's disease	Abdominal pain	(i) 7 out of 8 weeks (ii) Ongoing and episodic	(i) Intensity: 8–10 (ii) Nature: stomach spasms	No	(i) Acetaminophen (ii) Morphine (oral, about once a week)	(i) Hugs and cuddles (ii) Rocking	(i) *Challenge:* Crohn's disease has “complicated the picture,” a lot of potential causes of pain (ii) *Confidence:* confident she can assess child's pain (iii) *Expectation:* things are/will remain complicated

M8	Girl, 14 yrs, cerebral palsy, epilepsy, bilateral dislocated hips	(i) Dystonia (ii) Positional pain (sitting) (iii) Hip pain (iv) Moving and handling	(i) Frequency (not available) (ii) Regularity (not available)	(i) Intensity: not available (ii) Nature: not available	No	(i) Acetaminophen (ii) Ibuprofen (iii) Baclofen (iv) Codeine (as backup)	(i) Repositioning 1-2-hourly (ii) Bean bag (alternative position)	(i) *Challenge:* protecting child against future fractures and pain associated with these (ii) *Confidence:* confident about knowing signs of child's pain and what medication can be given (iii) *Expectation:* need to continue to be proactive about pain
